# Self-Assembled Nanoscale Materials for Neuronal Regeneration: A Focus on BDNF Protein and Nucleic Acid Biotherapeutic Delivery

**DOI:** 10.3390/nano12132267

**Published:** 2022-06-30

**Authors:** Yu Wu, Miora Rakotoarisoa, Borislav Angelov, Yuru Deng, Angelina Angelova

**Affiliations:** 1 CNRS, Institut Galien Paris-Saclay, Université Paris-Saclay, F-92290 Châtenay-Malabry, France; yu.wu@universite-paris-saclay.fr (Y.W.); miorantema@gmail.com (M.R.); 2 Institute of Physics, ELI Beamlines, Academy of Sciences of the Czech Republic, Na Slovance 2, CZ-18221 Prague, Czech Republic; borislav.angelov@eli-beams.eu; 3 Wenzhou Institute, University of Chinese Academy of Sciences, No. 1, Jinlian Road, Longwan District, Wenzhou 325001, China; dengyr@wibe.ac.cn

**Keywords:** neuroprotective assemblies, brain-derived neurotrophic factor (BDNF), nanomedicine for growth factor delivery, lipid nanoparticles, nanocarriers, nanofibers, biotherapeutics

## Abstract

Enabling challenging applications of nanomedicine and precision medicine in the treatment of neurodegenerative disorders requires deeper investigations of nanocarrier-mediated biomolecular delivery for neuronal targeting and recovery. The successful use of macromolecular biotherapeutics (recombinant growth factors, antibodies, enzymes, synthetic peptides, cell-penetrating peptide–drug conjugates, and RNAi sequences) in clinical developments for neuronal regeneration should benefit from the recent strategies for enhancement of their bioavailability. We highlight the advances in the development of nanoscale materials for drug delivery in neurodegenerative disorders. The emphasis is placed on nanoformulations for the delivery of brain-derived neurotrophic factor (BDNF) using different types of lipidic nanocarriers (liposomes, liquid crystalline or solid lipid nanoparticles) and polymer-based scaffolds, nanofibers and hydrogels. Self-assembled soft-matter nanoscale materials show favorable neuroprotective characteristics, safety, and efficacy profiles in drug delivery to the central and peripheral nervous systems. The advances summarized here indicate that neuroprotective biomolecule-loaded nanoparticles and injectable hydrogels can improve neuronal survival and reduce tissue injury. Certain recently reported neuronal dysfunctions in long-COVID-19 survivors represent early manifestations of neurodegenerative pathologies. Therefore, BDNF delivery systems may also help in prospective studies on recovery from long-term COVID-19 neurological complications and be considered as promising systems for personalized treatment of neuronal dysfunctions and prevention or retarding of neurodegenerative disorders.

## 1. Introduction

Neurodegenerative disorders have sophisticated etiology and represent a serious challenge for society [1,2,3,4,5,6]. Among the various risk factors, oxidative stress and chronic neuroinflammation (which can be due to viral infection or other causes) are involved in the pathogenesis of Parkinson’s disease (PD), Alzheimer’s disease (AD), Huntington disease (HD), and amyotrophic lateral sclerosis (ALS) [7]. These pathological conditions comprise the most common incurable neurodegenerative diseases (NDs), whose incidence and prevalence are growing. They are expected to surpass cancer with the second highest mortality rate [2,3]. PD is caused by the deterioration of dopaminergic neurons in the midbrain and is characterized by motor symptoms such as tremor, bradykinesia, and postural instability [4,5,6]. AD results from slow neuronal degeneration, which begins in the hippocampus and leads to the progressive loss of memory associated with a variety of neuropsychiatric and behavioral disorders [1]. 

Improved understanding of the multiple risk factors as well as prospective studies of the cognitive impairments, anxiety, depression, fatigue and sleep behaviour of COVID-19 survivors with new neurological complications (arising several months after long-term hospitalization) may contribute alternative therapeutic options to be developed against the long-term impact of COVID-19. Human severe acute respiratory syndrome coronavirus 2 (SARS-CoV-2) is neuroinvasive and may trigger acute or chronic neurological consequences following inflammation and oxidative stress [7,8,9,10,11]. Accumulating evidence has revealed that COVID-19 can damage not only the respiratory system but also other organs, including the brain and heart [12]. SARS-CoV-2 species have been detected in the cytoplasm of neurons in both the hypothalamus and cortex as well as in the cerebrospinal fluid of patients with COVID-19 [13]. The neuronal loss and damage caused by severe coronavirus infection have increased the number of vulnerable patients who may develop neurodegenerative disorders or long-term neuropsychiatric diseases after hospitalization [14,15,16,17]. Some literature reports have suggested that COVID-19 affects the progression of PD [18,19,20]. Others have emphasized the impact of COVID-19 on Alzheimer’s disease risk [21]. Recent studies have examined whether SARS-CoV-2 infection triggers the stimulation of caspase-2, caspase-3 and caspase-8 enzymes, the increased production of reactive oxygen species (ROS), and the diminishment of neurotrophic factor (e.g., brain-derived neurotrophic factor (BDNF)) levels [9,19,20,22,23].

The existing symptomatic treatments for NDs do not stop the spreading of the neuronal degeneration that is responsible for the progressive impairments in the patients’ daily lives [24,25]. Most of the proposed medications are oral formulations requiring high doses, associated with a subsequent high incidence of side effects. In general, the available medications against NDs only temporarily improve the disease symptoms by increasing the number of neurotransmitters in the brain. In AD, four drugs have been used for the treatment of the dementia phase, namely, the glutamate antagonist memantine and the cholinesterase inhibitors donepezil, rivastigmine, and galantamine [24]. PD patients have often received levodopa combined with a drug that delays the conversion of levodopa into dopamine until it reaches the brain [26]. Anticholinergics and other drugs, which mimic the role of dopamine in the brain, may help control tremor and rigidity. However, none of them stop the process of neuronal damage, which makes the disease ultimately fatal [25]. Currently, drug delivery technologies and alternative treatments that can prevent or delay neurodegeneration and promote neuroregeneration are urgently needed, especially for vulnerable patients in the long-term post-COVID-19 conditions. Studies have been initiated on targeting ND pathogenesis by macromolecular biotherapeutics, including antibodies, growth factors, nucleic acids, and enzymes [27,28,29]. The designed neuroregenerative strategies aim to repair neuronal damage. However, the major challenge for clinical applications is because the brain is protected by the blood–brain barrier (BBB), through which only specialized small-molecule drugs can pass [30].

In addition to our previous reviews [31,32], in this work, we provide an overview of more recent examples of neurotrophic protein and peptide drug administration as well as of nucleic acid utilization for accelerating the regeneration of damaged neurons. We emphasize the biomimetic assemblies and nanoscale structures, which have shown promise for safe and more efficient drug delivery to the central and peripheral nervous systems. Recent nanotechnology strategies for the delivery of growth factors by liposomes, solid lipid nanoparticles (SLNs), polymeric nanoparticles, hydrogels, or nanofibers are outlined, with a focus on the outcomes of BDNF-loaded nanoparticles and nanofibers in neuronal regeneration trials.

## 2. Biomolecule Delivery in Neuroregeneration Strategies

The diverse side effects found with conventional ND treatments using small molecule compounds have encouraged research on alternative therapeutic modalities in drug delivery aimed at neuroregeneration. In principle, regeneration of neurons can be stimulated by either enhancing endogenous neurogenesis upon the administration of growth factors or by the transcription of genes involved in neuronal survival [27,33,34].

### 2.1. Neuroprotective Biomolecules and Nucleic Acids under Current Investigation

#### 2.1.1. Neurotrophic Factor Protein-Based Therapies

Neurotrophic factors (NTFs) are a family of biomacromolecules (large peptides or small proteins) that support the growth, survival, and differentiation of developing and mature neurons by protecting them from injury and neurotoxins [34,35]. Nerve growth factor (NGF) was the first NTF discovered by Levi-Montalcini [36]. Subsequently, the neuroprotective functions of several other NTFs have been reported over the years [33,37,38,39,40,41,42,43,44,45,46]. They have been categorized into three main families: (i) the neurotrophin family, including NGF, BDNF, neurotrophin-3 (NT-3), and neurotrophin-4 (NT-4); (ii) the glial cell line-derived neurotrophic factor (GNDF) family, e.g., GDNF, neurturin (NRTN), artemin (ARTN), and persephin (PSPN); and (iii) the neuropoietic cytokines, e.g., ciliary neurotrophic factor (CNTF), leukemia inhibitory factor (LIF), and cardiotrophin (CT-1). Other proteins, such as fibroblast growth factor-1 and -2 (FGF-1 and FGF-2) and platelet-derived growth factor (PDGF), as well as polypeptides, including pituitary adenylate cyclase-activating peptide (PACAP), insulin-like growth factor 1 (IGF-1), human neuropeptide substance P, macrophage colony-stimulating factor (M-CSF), and granulocyte-macrophage colony-stimulating factor (GM-CSF), can also play a role as NTFs [47,48,49,50,51,52,53,54,55,56,57,58].

A novel family of unconventional NTFs, cerebral dopamine neurotrophic factor (CDNF) and mesencephalic astrocyte-derived neurotrophic factor (MANF), which are both structurally and mechanistically distinct from the other growth factors, have shown neurorestorative effects in animal models of PD [33]. These biotherapeutics localize to the lumen of the endoplasmic reticulum (ER) and likely modulate the unfolded protein response (UPR) pathway. Intermittent monthly bilateral intraputamenal infusions of CDNF have recently been tested in a randomized placebo-controlled phase I–II clinical trial in PD patients [33].

Studies of an AD rat model with amyloid-β-induced memory loss have demonstrated that granulocyte colony stimulating factor (GCSF), an endogenous neuronal hematopoietic factor protein, improves memory and neurobehavioral functions [39]. GCSF exerted neuroprotective activity associated with significant memory improvements, increased levels of antioxidant enzymes and total RNA expression in the brain, and reduced lipid peroxidation and acetylcholinesterase levels. In addition, GCSF induces neurogenesis, as evidenced by the increased number of progenitor CD34+ cells in the brain [39]. Clinical trials using GCSF for the treatment of AD and stroke have already been carried out [55,56,57]. The advantages of GCSF, as a good candidate for clinical trials in NDs, also include its capacity for crossing the BBB and its strong anti-apoptotic activity.

Several clinical trials have been conducted to examine the capacity of GDNF, NRTN and PGDF to rescue degenerating dopaminergic neurons in the substantia nigra and their axon terminals in the striatum [44,54]. GDNF has been studied as a candidate in clinical trials of PD considering its neurorestorative effects established in PD animal models [51,58]. The performed in vitro and in vivo studies with PD models have demonstrated the neuroprotective and neurorestorative effects of GDNF on midbrain dopaminergic neurons [51,52,53,54]. Unlike GCSF, the penetration of GDNF in the brain is strongly limited. Therefore, various strategies have been undertaken for GDNF delivery to the dopamine-depleted brain, e.g., implantation of microspheres, transfection by viral vectors, or ventricle and intraputaminal infusion of the protein [58,59,60]. The delivery of BDNF by nanoparticles and other biomimetic nanoscale assemblies will be presented in a separate section below.

#### 2.1.2. siRNA-Based Therapy

Emerging strategies for the prevention or treatment of NDs are being developed based on selective silencing of mutant alleles. This approach aims to directly arrest the causative mutant genes for neurodegeneration [61]. RNA interference (RNAi) regulates the expression of genes by controlling the synthesis of proteins via a post-transcriptional gene-silencing mechanism. Long double-stranded RNA sequences are cleaved by the cytoplasmic enzyme Dicer into fragments (21–23 nucleotides long) called small interfering RNAs (siRNAs). siRNA is incorporated into a protein complex called the “RNA-induced silencing complex”, and then the sense strand of the siRNA is cleaved. The antisense strand guides the RNA-induced silencing complex to bind with a messenger RNA (mRNA), which is complementary to the antisense strand and degrades it. An important advantage of RNAi over small-molecule and protein therapeutics is that mutant alleles can be targeted with RNAi. In principle, any transcript that encodes a protein that causes or contributes to a disease can be targeted by RNAi [62]. Therefore, a major advantage of sequence-based targeting technologies is the ability to design precisely targeted biotherapeutics for almost any target sequence (coding or noncoding), regardless of the function of the gene product [63].

The therapeutic potential of RNAi in AD has been demonstrated through allele-specific gene silencing by short-hairpin RNA (shRNA) [62]. An anti-APPsw shRNA was delivered by the recombinant adeno-associated virus to the hippocampus of AD transgenic mice (APP/PS1) to selectively suppress mutant APP. No neuronal toxicity was detected in short- and long-term transduction experiments with the viral vector. Intravenously injected rabies virus glycoprotein (RVG)-targeted exosomes have specifically delivered siRNA to neural cells in the mouse brain. Strong mRNA (60%) and protein (62%) knockdown of BACE1 was achieved without noticeable immune stimulation. CBP-1 (acetyltransferase enzyme) has been inhibited by RNAi to evaluate the age-dependent mortality rate for 30 drugs used for protection of mammalian neurons. The genes of interest, which may be more specifically involved in the tau phosphorylation pathways in AD, are DYRK1A and AKAP13 [62].

Several obstacles remain for the clinical development of RNAi-based therapeutics [63]. The delivery issue represents a major challenge, as siRNA should be transferred to specific target sites, and the potential off-target effects should be taken into consideration as well. AD is a multifactor and genetically heterogeneous disorder. It cannot be treated by a single siRNA sequence. Therefore, new strategies should be envisioned to formulate the various RNAi components and successfully deliver them to the target sites.

### 2.2. Therapeutic Delivery Approaches for Neuroprotective Biomacromolecules

#### 2.2.1. Invasive versus Noninvasive Administration of Carrier-Free Biomolecules

The major reason for the limited effect of therapeutic biomacromolecules (therapeutic peptides or proteins) in clinical trials has been attributed to the presence of the BBB [27,30]. Local delivery to the brain has been suggested via stereotactic cerebral injection or intracerebral infusion [32]. The problem of this approach is the difficulty in determining the most appropriate doses of each compound. For instance, intracerebral neurotrophic factor administration has shown no improvement of motor symptoms in PD (owing to the difficulty for the drug to cross the blood–brain barrier) and thus represents its limited efficacy in clinical trials [64]. Therefore, different approaches for biomolecule delivery are required to increase bioavailability [65,66,67,68].

**Figure 1:**
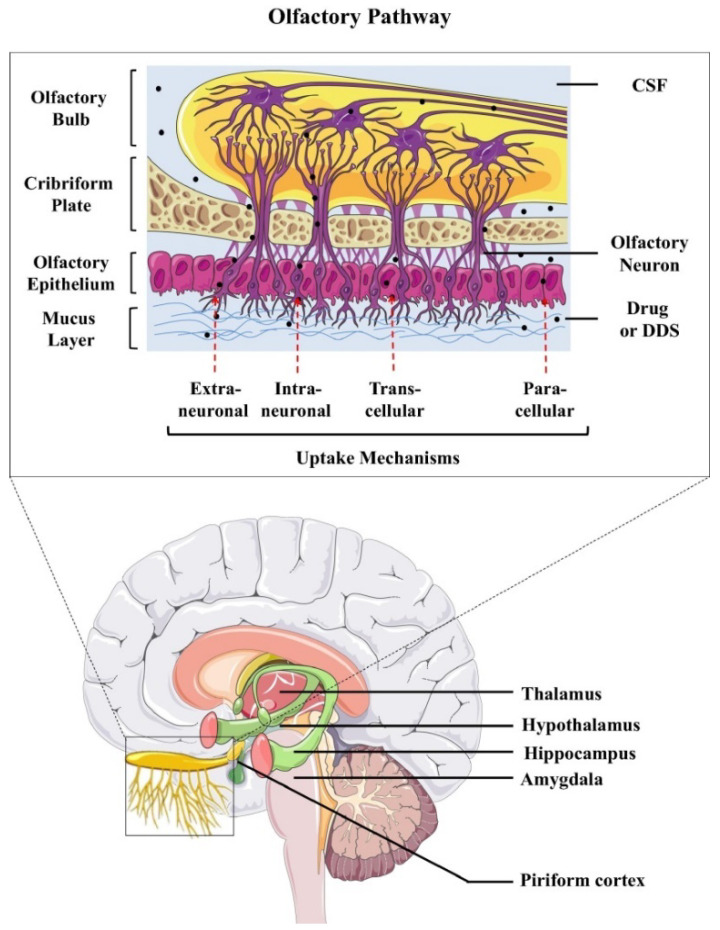
Uptake mechanisms involved in the transport of therapeutic proteins from the nasal cavity directly to the brain via the olfactory nerve pathway (Reprinted with permission from Ref [70]. Copyright 2018 Elsevier).

A direct route to reach the brain without going through the BBB is the nasal-to-brain delivery route (Figure 1) [69,70]. Intranasal drug administration avoids hepatic first-pass metabolism and has been considered a safe, noninvasive route [71,72,73]. In this method, the therapeutic drug, which is applied into the nasal cavity, can penetrate the central nervous system (CNS) via the olfactory and/or trigeminal nerves [73]. Different models have been used to evaluate nasal drug absorption both in vitro and in vivo [70,73,74,75]. Some biomolecules, such as CNTF, BDNF, and NT-4/5, have been successfully delivered to the hippocampus and cerebral cortex of rats. Quick absorption of BDNF has been observed due to the interaction of BDNF molecules (exposing cationic surface charges) and the nasal mucosa (negatively charged) [75].

#### 2.2.2. Gene Delivery

Another strategy to alter local protein expression is based on gene delivery [76,77]. Several clinical trials have been performed to examine the capacity of neurotrophic factors to rescue degenerating neurons by viral vector-mediated gene delivery to the brain [76,77,78,79,80]. A cationic nanocarrier functionalized by dexamethasone and cell-penetrating peptides increased BDNF expression upon BDNF DNA delivery [77]. Many authors have demonstrated the tolerability of gene delivery to PD patients (e.g., intraputaminal injections of adeno-associated virus serotype 2-neurturin (CERE-120)) in a phase I open-label clinical test [78,79,80]. Although these gene therapy approaches have been shown to be safe, their efficacy in phase II clinical trials has been considered insufficient [80].

#### 2.2.3. Carrier-Mediated Delivery Employing Different Nanoscale Materials

Recent research has focused on the development of neurotrophin delivery systems that can provide a safe and efficient neurotrophic supply over the long term [81,82,83,84,85,86,87,88,89]. It has been of special interest to combine such systems with implants, i.e., to explore implant-coupled drug delivery [81,82,83]. An encapsulated cell biodelivery (ECB) device has been demonstrated to be an efficient method to improve NGF levels in AD patients [89]. Other promising approaches have comprised electrode coating materials [82] as well as carrier systems such as hydrogels [83,84], microspheres [85], nanotubes [47], mesoporous silica supraparticles [86], or nanoparticles [87,88].

## 3. Nanoscale Materials for Stimulation of Neurogenesis and Neuroregeneration

**Figure 2:**
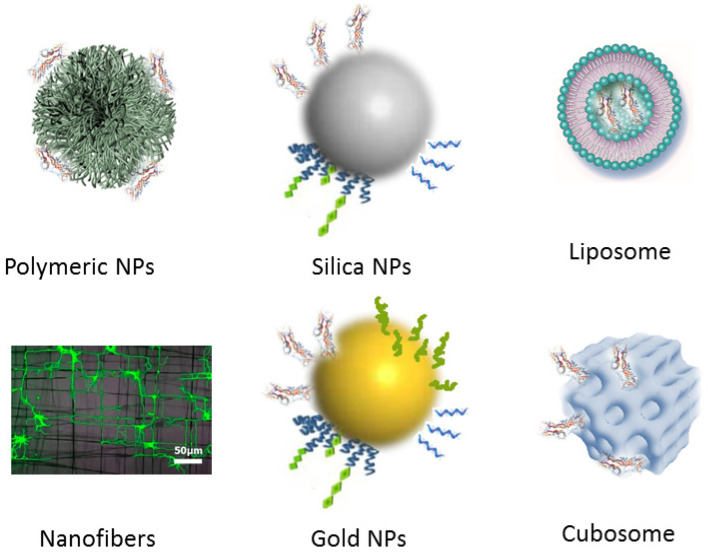
Schematic presentation of functionalized nanoparticles (NPs) and nanoscale materials for targeted drug delivery: polymeric nanoparticles of biodegradable nature (PLGA or PLGA-PEG-PLGA), inorganic silica and gold nanoparticles functionalized with surface-anchored ligands, nanofibers for sustained release of neurotrophic compounds, and lipid-based self-assembled liquid crystalline nanocarriers (liposomes and cubosomes) for protein and gene delivery.

For a long time, the availability of effective treatments against NDs has been restricted not only by the brain structure, which is protected by the BBB, but also by the high cost of CNS drug development. Extended time periods have been needed to establish whether investigational treatment may truly affect disease progression [90,91,92,93,94]. As most growth factor proteins do not cross the BBB, they must be delivered intracranially [31]. Various reports have emphasized that the efficient diffusion of NTFs in brain tissue is of crucial importance [95,96,97,98,99,100,101,102,103,104,105]. From this perspective, nanoparticles have been largely investigated for neurotrophic factor delivery to improve penetration and diffusion in the brain [32,106,107]. In recent years, different types of nanoparticles [108,109] have been exploited to enhance drug delivery efficacy towards neurogenesis and neuroregeneration, e.g., polymeric nanoparticles, silica nanoparticles, nanofibers, gold nanoparticles, liposomes, cubosomes, and other lipid-based liquid crystalline nanoparticles (Figure 2).

### 3.1. Functionalized Nanoparticles for Brain-Targeted Drug Delivery

**Figure 3:**
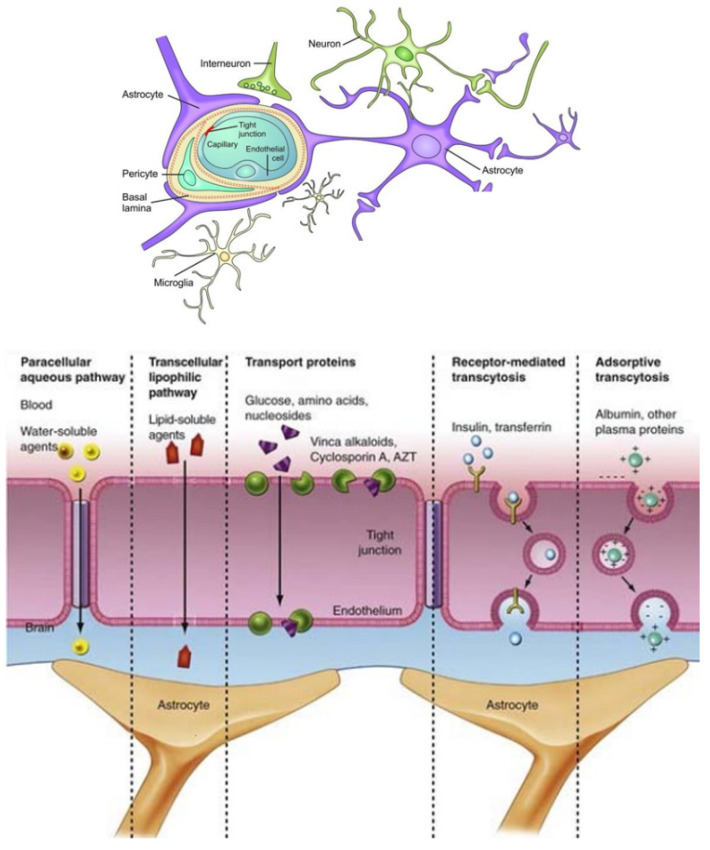
Schematic presentation of the microstructure of the blood–brain barrier (BBB) and possible mechanisms of biomolecule passage to the central nervous system (Reprinted from Ref [128]. MDPI Open Access 2019).

Transport of therapeutic biomolecules by nanoparticles through the BBB and cellular membranes can increase the chances for more efficient therapy against NDs [110,111,112,113,114,115,116,117,118,119,120,121,122,123]. The nonselective distribution of drug compounds in the brain hampers the effective treatment of neurodegenerative disorders, as serious side effects may be caused with regard to normal brain function. Functionalized nanoparticles have been intensively studied for improving the permeability of the BBB [124,125,126]. An important advantage is that the nanosized particles can be functionalized for targeted drug delivery to PD or AD lesions [127] as well as for receptor-mediated transcytosis [128] (Figure 3).

A recent study of SLNs for drug delivery across the BBB explored the chemical modification by borneol (BO) of dioleoyl phosphoethanolamine (DOPE), which is one of the lipid constituents employed [129]. The borneol-modified solid lipid nanoparticles (BO-SLN/CM) displayed lower cytotoxicity, better cellular uptake, and enhanced BBB permeability compared to conventional SLNs. Whereas the control group of nonmodified SLNs accumulated in the lungs, the BO-SLN/CM considerably penetrated the brain. Thus, the synthesized BO-SLN/CM has emerged as a promising lipid-based system for targeted delivery across the BBB [129]. Other recent in vitro and in vivo reports have investigated dual-functionalized nanocarriers, which have demonstrated brain-targeting effects linked with the use of cholesterol-polyethylene glycol (PEG) and poly(ethylene glycol)-poly(lactide) [130].

PEGylated liposome and cubosome liquid crystalline particles have shown a capacity for delivering different proteins or genetic materials across the BBB [131,132]. Functionalized liposomes and solid lipid nanoparticles, characterized by a high affinity for the amyloid beta (Aβ) neurotoxic peptide, have been broadly considered in AD research [133]. A dual-functionalized nanoparticle-based drug delivery system was designed using a PEGylated poly(lactic acid) (PLA) polymer. Two targeting peptides, TGN and QSH (screened by phage display), have been conjugated to the surface of the nanoparticles. The TGN functionality was suitable for targeting ligands at the BBB, whereas the QSH had a good affinity for the Aβ1-42 sequence, which is a main component of amyloid plaques [134].

### 3.2. Neuron-Targeted Biomolecule Delivery by Nanocarriers

#### 3.2.1. Nanoparticles for Protein Delivery

Innovative neuron-targeted delivery systems are urgently needed and are being developed based on nanocarriers [135,136,137,138,139,140,141,142]. For the delivery of therapeutic proteins, the designed nanocarriers should enable efficient loading and retention of the entrapped protein biomolecules in the self-assembled nanoscale reservoirs. These carriers should be stable in the biological milieu and ensure suitable release profiles for the functional proteins to interact with their receptors at the sites of action. Notably, surface-modified nanoparticles may increase the permeability of the BBB [135,136]. Growth factors (GFs) and laminins are two examples of biomolecules involved in regeneration processes [33,34,35,36]. GF proteins play an important role in various events, such as cellular proliferation and differentiation. Due to their short half-life, different delivery strategies have been proposed to minimize GF protein degradation in the circulation [137,138]. Polylactic acid (PLA), polylactic-co-glycolic acid (PLGA), and polyglycolic acid (PGA) have been used for growth factor delivery as commonly exploited synthetic biodegradable polymeric matrices in bone regeneration [137,138]. In fact, PLA and PLGA nanoparticle systems have been exploited in experiments for both short- and long-term delivery of biomolecules.

Inorganic mesoporous silica nanoparticles (MSNs), containing immobilized bone growth factors, have been shown to facilitate osteogenic differentiation of human mesenchymal stem cells (hMSCs) [139]. Based on in vitro experiments, Prades et al. reported that gold nanoparticles (AuNPs) conjugated with a CLPFFD peptide (AuNP-CLPFFD) can destroy toxic β-amyloid aggregates (Aβ) [140]. In this case, CLPFFD was chosen as a β-sheet breaker peptide, which recognizes aggregated Aβ. To enhance the permeability in the brain, a second peptide (THR) has been introduced for targeting a receptor present at the neuronal cell membranes. Remarkably, the created AuNP-THR-CLPFFD complex has been established to accumulate in the central nervous system. It should be concluded that AuNPs have the potential to deliver therapeutic peptides or proteins to the brain through certain conjugation strategies [140].

**Figure 4:**
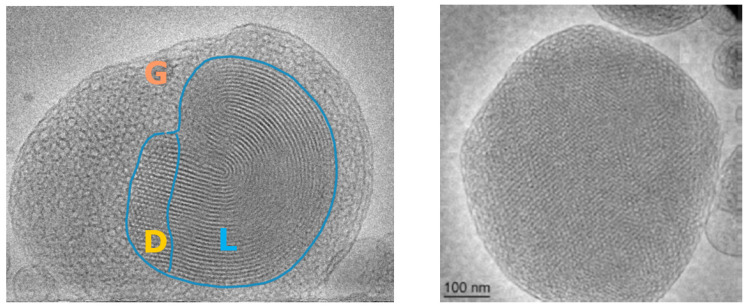
Cryo-TEM image of multicompartment cubosome particles loaded with the neurotrophic protein BDNF. (Reprinted with permission from Ref [144]. Copyright 2020 American Chemical Society) BDNF is a water-soluble protein, which interacts with the lipid bilayer, changes the membrane curvature, and induces multiphase domains within the self-assembled lipid membrane particles. L—denotes lamellar phase domain, D—double diamond type cubic phase domain, and G—gyroid type cubic phase domain.

Chitosan nanocarriers have been used for the delivery of an Aβ antigen [141]. Aβ antigen, which was injected into the caudal vein of mice, was subsequently detected in the brain. The obtained results indicated that chitosan nanocarriers can increase the permeability of the BBB and successfully deliver proteins in the mouse brain [141]. Recently, the peptide H102 (HKQLPFFEED), which is another β-sheet breaker, has been found to improve the spatial memory impairments of mice [142]. This finding presents another opportunity for AD treatment. Zhang et al. described the delivery of the H102 peptide to the brain of an AD mouse model by PEG–PLA nanoparticles [142]. Therefore, PEG–PLA nanoparticles may also be considered an opportunity for peptide or protein drug delivery in ND models. Targeted albumin nanoparticles modified by apolipoprotein E have shown strong cellular uptake in the mouse brain [143]. Lipid-based cubosome nanoparticles have been designed for BDNF loading [144]. The multicompartment self-assembled organization of the BDNF-loaded nanocarriers (cubosomes) was revealed by cryo-TEM imaging (Figure 4).

Nanoparticles are promising carriers for the delivery of peptide and protein drugs by intranasal administration [65,66,67,68,69,72,73,74,75,91,120,122]. Vasoactive intestinal peptide (VIP), which has anti-inflammatory activity, is a 28-amino acid neuropeptide. Its clinical effect is essentially limited due to the rapid degradation of VIP in the blood circulation. Gao et al. encapsulated peptide (VIP) molecules in functionalized PEG-PLA nanoparticles (VIP-NPs) [145]. The uptake of VIP-NP in the brain was achieved by intranasal administration. Subsequently, basic fibroblast growth factor (bFGF) has been entrapped in functionalized polyethylene glycol-polylactide-polyglycolide (PEG-PLGA) nanoparticles [146]. Enhanced spatial learning and cognitive function effects have been reported following the intranasal administration of bFGF-NPs [146].

#### 3.2.2. Nanoparticles for Gene Delivery

**Figure 5:**
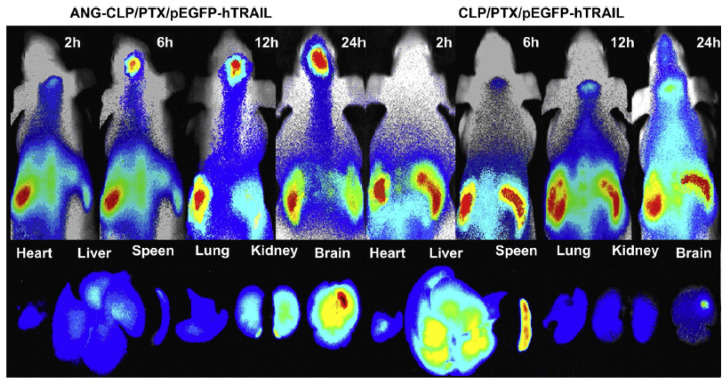
Tumor-bearing brain accumulation of ANG-CLP/PTX/pEGFP-hTRAIL liposomes (i.e., Angiopep-2-modified liposome assemblies loaded with pEGFP-hTRAIL and PTX were visualized by real-time in vivo fluorescence imaging of intracranial U87 MG glioma tumor-bearing nude mice after intravenous injection. (Reprinted with permission from Ref [152]. Copyright 2020 Elsevier).

Nucleic acid delivery may directly regulate the causative genes of diseases with limited side effects of gene therapy [147,148,149,150]. The development of efficient nonviral gene delivery systems remains a key challenge for the clinical application of RNA interference (RNAi) therapeutics in neurological diseases. To deliver siRNAs to brain neuronal cells, nonviral gene carriers are required to cross the BBB and overcome intracellular membrane barriers by avoiding lysosomal degradation. Functionalized nanoparticles have been proposed as a promising strategy to protect RNA from degradation [151]. The nanoparticle surface can be modified by chitosan or by different peptides, which have the ability to interact with brain endothelial cells via the receptor-mediated transcytosis (RMT) mechanism and then target neuronal cells. Sun et al. demonstrated a dual-targeting effect via angiopep-2-modified cationic liposomes (ANG-CLPs), which have been designed for the codelivery of a therapeutic gene encoding human tumor necrosis factor-related apoptosis-inducing ligand (pEGFP-hTRAIL) and paclitaxel (PTX) [152] (Figure 5).

Park et al. developed a nanoparticle system (R-PEG-PMT/siRNA) composed of siRNA and a nonviral vector, poly(mannitol-co-PEI) gene transporter (PMT), which has been modified by a rabies virus glycoprotein (RVG) peptide fragment [147]. The RVG peptide has been widely investigated in CNS targeting and penetration. The in vitro BBB penetration study confirmed that the internalization of the RVG-PEG-PMT/siRNA complex was enhanced compared to that of the control group (PEG-PMT/siRNA). The intravenously injected RVG-PEG-PMT/siRNA complex reduced BACE1 (beta-site APP cleavage enzyme 1) in mice. The opportunities to use RVG-PEG-PMT/siRNA assemblies in AD treatment have been outlined considering that BACE1 regulates the levels of the pathogenic amyloid-beta Aβ42 (42-amino acid isoform) [147]. To enhance the biotherapeutic targeting effect, Liu et al. proposed a multifunctional nanoparticle system for BACE1 siRNA delivery [148]. The chosen D-peptide has been proven to decrease tau fibril formation and ameliorate AD symptoms. Both an RVG peptide and a D-peptide have been grafted to a dendrigraft poly-L-lysine (DGL) nanoparticle surface. The penetration and BACE1 silencing effects have been confirmed in in vitro and in vivo studies [148]. Another work reported the delivery of BACE1 siRNA to neuronal cells using functionalized PEG-PLGA nanoparticles [151]. Wang et al. synthesized a CGN peptide sequence (d-CGNHPHLAKYNGT) whose targeting capacity has been examined both in vitro and in vivo [151]. The CGN-modified nanocomplexes inhibited 50% of BACE1 expression in PC12 cells and enhanced the learning ability of AD animal models. These results have indicated the potential of the investigated nanosystems for neuron-targeted gene delivery towards AD treatment.

Several recent studies have focused on the delivery of neurotrophic genes to neuronal cells to regulate the local concentration of expressed neurotrophins. Arora et al. reported mannose- and cell-penetrating peptide (RVG)-modified liposomes for transferring the BDNF gene to neuronal cells [149]. BDNF levels in the brain increased after the intravenous injection of the liposome complexes in mice. The recognized efficacy of the p11 gene for depression has been investigated with nanoscale carriers. Gandhi et al. designed a liposome system using synthetic lipids to make gene delivery safer [150]. For targeting purposes, the liposome surface has been modified with an insulin-like growth factor II (IGF-II) monoclonal antibody. As an outcome, the liposomal complex has been characterized by improved stability and distribution in the brain [150].

### 3.3. Nanomaterials Promote Neuroregeneration by Targeting the Extracellular Environment

**Figure 6:**
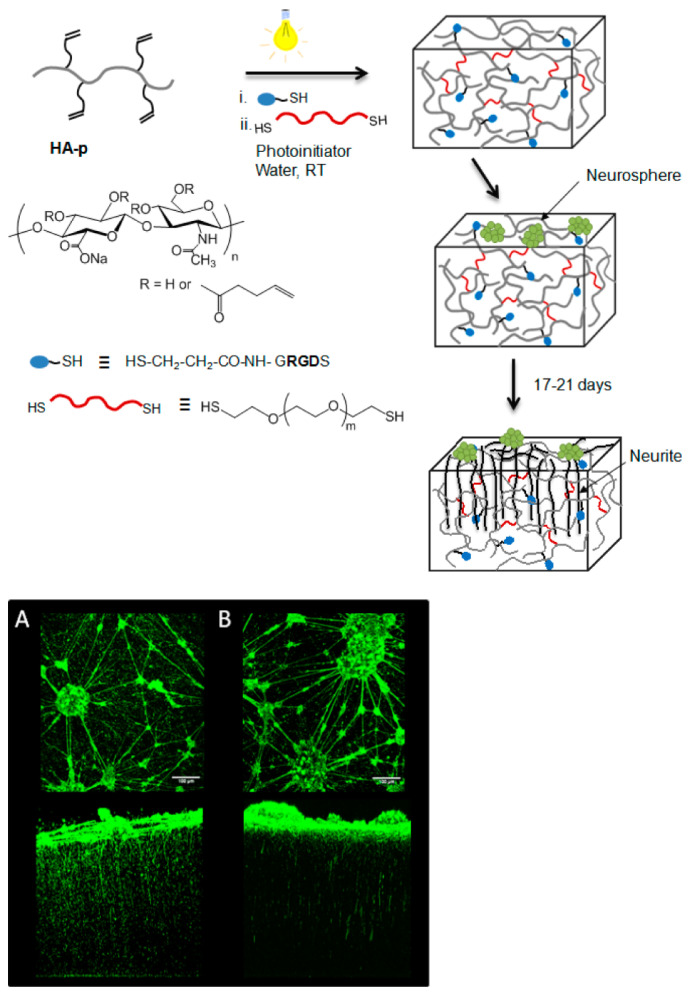
(Top panel) Scheme of the preparation of hyaluronic acid (HA)-based hydrogels functionalized with RGD ligands for central nervous system (CNS) regeneration. (Bottom panel) 3D two-photon microscopy images of neurite outgrowth (β3 tubulin staining) at day 21 after plating hippocampal neural progenitor cells on the surface of hydrogels with a storage modulus of 400 Pa (**A**) or 800 Pa (**B**). (Reprinted with permission from Ref [153]. Copyright 2016 American Chemical Society).

Hydrogel nanoscaffolds can facilitate neuronal growth and neuroregeneration by creating an artificial extracellular matrix (ECM). A hyaluronic acid (HA)-based ECM platform, which imitates brain characteristics, has been prepared [153]. A cell-adhesive peptide, arginine-glycine-aspartic acid (RGD), which is the most common peptide motif for cell adhesion in ECM, has been linked to HA hydrogels (Figure 6). Two-photon microscopy images have demonstrated that the modified HA hydrogel promotes neural outgrowth behavior and differentiation [153]. Neural stem cells (NSCs) play an important role in neurogeneration, which can produce new oligodendrocytes, astrocytes and neurons. It has been established that a hepatocyte growth factor (HGF)-loaded hydrogel can promote NSCs in vitro [154].

### 3.4. Multifunctional Nanomaterials Promoting Neuroregeneration

The advantages of a combination of nanomaterials for neuronal tissue regeneration and improved control of drug release kinetics have been implemented in peripheral nerve generation strategies [155,156]. Nanofibrous scaffolds composed of a natural polymer (SF) and a synthetic polymer (P(LLA-CL)) were fabricated for the encapsulation of NGF [157]. Sustained release of NGF was achieved within 60 days. Peripheral neuroregeneration effects have been observed in rats [157].

## 4. BDNF Delivery by Nanocarriers and Nanoscale Materials in Neuronal Diseases

**Figure 7:**
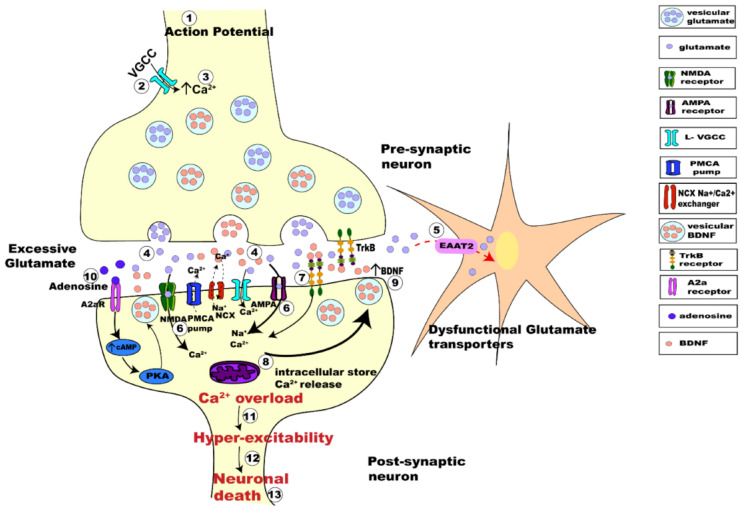
Scheme illustrating BDNF transport by dense core vesicles and its release activating neurotrophic BDNF-TrkB signaling, which interplays with glutamate-induced excitotoxicity activities in synapses. (Reprinted from Ref [158]. Frontiers Open Access 2019).

BDNF is a secretory neurotrophic protein that plays a key role in the neurogenesis and survival of neuronal cells [41]. There are 3 different forms of BDNF in mammals: prepro-BDNF, pro-BDNF, and mature BDNF [40]. BDNF is a high-affinity ligand for tropomyosin-related kinase receptor (TrkB). It binds to the receptor and activates the MAPK, PI3K and PLC-γ signaling pathways, which are implicated in neuroprotective and neuroregenerative effects [158]. The BDNF mechanism is used as an emerging targeted strategy in neurorepair [29,158,159]. Figure 7 shows the BDNF-TrkB signaling involved in synaptic transmission. The neurotrophic protein is localized within dense core vesicles, which are responsible for the transport and release of BDNF [158].

BDNF levels have been established to significantly decrease in several CNS diseases [41,160]. Further to the role of SARS-CoV-2 infection in AD progression associated with oxidative stress and neuroinflammation [21], recent research has confirmed that coronavirus infection may essentially influence BDNF expression levels and thus may impair BDNF/TrkB signaling [161,162,163,164]. It has been well documented that decreased BDNF levels present a serious risk factor for neurodegeneration [41,43,164]. Many studies have demonstrated the beneficial effects of neurotrophic BDNF delivery in neuronal pathologies towards the promotion of neural differentiation and survival and the amelioration of memory and learning capacities (among various other features) [31,32,44,45,160]. Therefore, BDNF delivery carriers are receiving increasing interest for the translation of nanomedicine into clinics. In the following, we summarize the recent devopments of nanoscale carriers of BDNF, which show potential for exploration also in the research with vulnerable post-COVID-19 patients.

### 4.1. BDNF Protein Delivery by Nanocarriers to Neurons

Harris et al. performed a polyion complexation of BDNF with PEG(5 kDa)-PGA(9 kDa) diblock copolymer to protect BNDF from rapid degradation in the circulation [165]. The obtained formulation of BDNF nanocomplexes increased BDNF levels in mice and exerted a therapeutic effect on stroke [165]. To improve the stability of BDNF in the presence of serum, BDNF was stabilized by transient hydrogen bonding and cooperative electrostatic interactions using the anionic block copolymer poly(ethylene glycol)-b-poly(l-glutamic acid) (PEG-PLE). This nanoformulation ameliorated the stability of neurotrophin in the circulation without changing the affinity interaction between BDNF and its receptor [166]. Various other hydrogel-based scaffolds have been investigated for BDNF encapsulation and delivery with beneficial outcomes as well [167,168,169,170,171]. PEGylated liposome nanoparticles can serve as efficient nanocarriers to the brain [172]. Xing et al. employed a PEG-conjugated liposomal BDNF vector with a cytomegalovirus promoter (pCMV), which enabled increased BDNF expression [172]. Nanofibers have been widely used as an excellent matrix to help achieve sustained release of BDNF [173]. A cochlear implant including BDNF-loaded nanoporous silica nanoparticles released BDNF over 80 days [174].

### 4.2. Nanoparticles Modified by BDNF-Derived Peptides for Drug Delivery to Neurons

**Figure 8:**
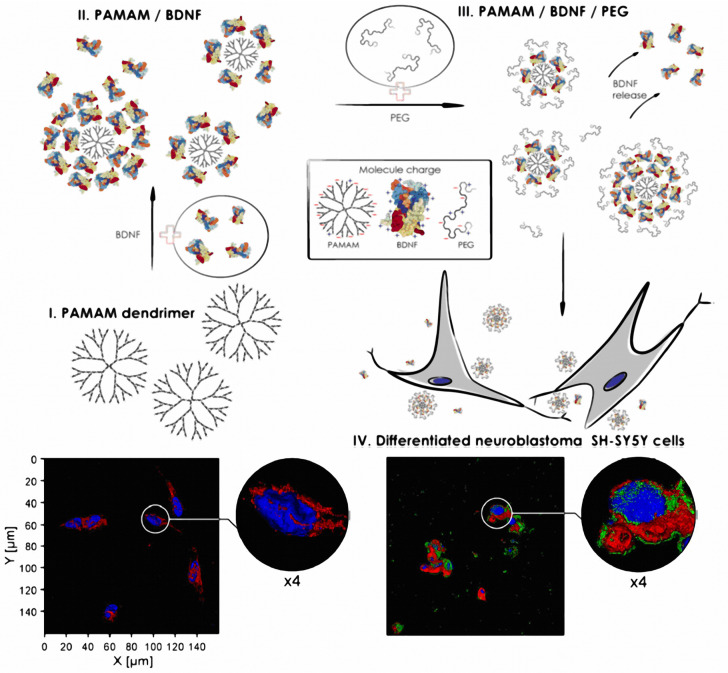
Scheme of the preparation of PEGylated PAMAM-based nanoparticles containing BDNF and images showing the cellular localization of the nanoparticles in SH-SY5Y cells. The panel on the left presents the control group. The panel on the right presents the cells after 24 h of exposure to BDNF-PAMAM-AF488-PEG. The nanoparticles are observed in green, and the cells are costained with WGA-Texas Red-X (red) and DAPI (blue). (Reprinted with permission from Ref [176]. Copyright 2020 Springer Nature).

The recognition mechanism of BDNF ligands has been used as a targeted strategy to the CNS [175]. Xu et al. demonstrated the internalization of PEG-PCL nanoparticles, whose surface was decorated by a BDNF-derived (IKRG) peptide, into neuronal cells [175]. The tetrapeptide (IKRG) amino acid sequence has been shown to mimic the function of BDNF in targeting TrkB receptors, which are abundant in neurons [175]. Enhanced uptake of peptide-modified PEG-PCL nanoparticles has been observed in TrkB-positive PC12 cells but not in TrkB-negative HeLa cells [175]. Dąbkowska et al. successfully delivered BDNF to neuronal SH-SY5Y cells via PEGylated poly(amidoamine) dendrimer (PAMAM) nanoparticles [176]. The BDNF-loaded nanoparticles were stabilized by electrostatic interactions (Figure 8). The studied BDNF-PAMAM-AF488-PEG nanoparticles have been characterized by slow release of the therapeutic agent and strong interaction with the cell membrane surface [176].

A nanofiber hydrogel has been formulated with a mixture of two peptides, one of which is a BDNF mimetic peptide [177]. The purpose has been to promote the promyelination of Schwann cells and the adhesion and proliferation of endothelial cells. RKKA_D_P is a BDNF mimetic peptide that self-assembles in water and forms a hydrogel network [177]. Edelbrock et al. reported that BDNF mimetic peptide can activate BDNF-TrkB signaling as well as other downstream signaling cascades capable of promoting neuronal cell infiltration and functional maturation [178]. The regenerative efficacy, maturation of nerve fibers, and vascularization effect have also been confirmed in vivo.

### 4.3. BDNF Gene Delivery by Nanocarriers

BDNF gene delivery has been a promising strategy for targeting peripheral neuronal cells. Chitosan-based nanocarriers, which are biodegradable and biocompatible, have been demonstrated as suitable for the condensation and compaction of nucleic acids as well as for preventing BDNF endonuclease degradation [179]. A polymeric nanoparticulate carrier composed of trimethyl chitosan (TMC) has been used for the transfection of therapeutic BDNF plasmid DNA [179]. Significantly increased BDNF levels and subsequent neuronal regeneration have been observed in mice compared to the non-treated group [179]. The performed study demonstrated the role of the targeted nanoparticles in the efficacy of BDNF gene delivery.

### 4.4. BDNF Delivery by Hybrid Systems and Scaffolds for Tissue Engineering

**Figure 9:**
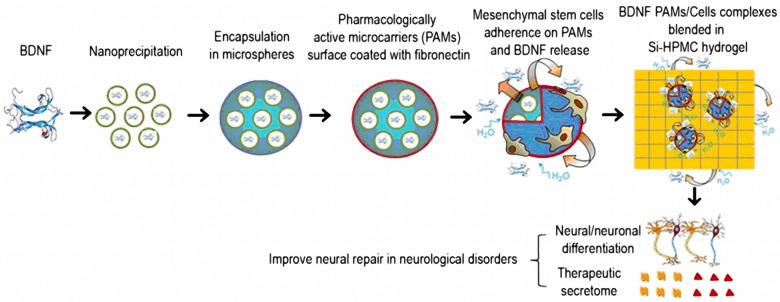
Strategy of BDNF delivery using pharmacologically active microcarriers (PAMs) coated with fibronectin and embedded in a hydrogel scaffold. (Reprinted with permission from Ref [171]. Copyright 2017 Elsevier).

Tissue engineering has been extensively investigated for the purposes of long-term neurotrophin delivery [180,181]. The local delivery of BDNF using mesenchymal stem cells (MSCs) has provided a continued release of BDNF for 14 days and a recovery of functional activity in an animal spinal cord hemisection [182]. Schwann cell (SC)-seeded alginate hydrogels have been administered to the spinal cord lesion site. The sustained release of BDNF facilitated the axonal growth and pro-regenerative effect of the alginate gels seeded with SCs [183]. A cochlear implant was fabricated for BDNF, GDNF, and laminin delivery using a hydrogel (loaded with fibrin and collagen, which was covered by human adipose-derived stem cells [184]). The expression of BDNF has been detected over a week with a produced quantity (up to 2.59 ng/mL in the supernatant) that has been sufficient for neurotrophic effects [184]. Fibronectin-coated pharmacologically active microcarriers were prepared for the encapsulation of BDNF by nanoprecipitation of poloxamer with glycofurol in NaCl medium (Figure 9). Human mesenchymal marrow-isolated adult multilineage-inducible (MIAMI) stem cells have been attached to the surface of the microcarriers to enhance the secretion of several growth factors, including BDNF. The sustained release of BDNF over 40 days promoted neuronal repair [171]. 

The therapeutic outcomes observed with recently described neurotrophin carrier systems are summarized in Table 1.

## 5. Nanoscale Assemblies of Bioactive Lipids Offering Therapeutic Opportunities

**Figure 10:**
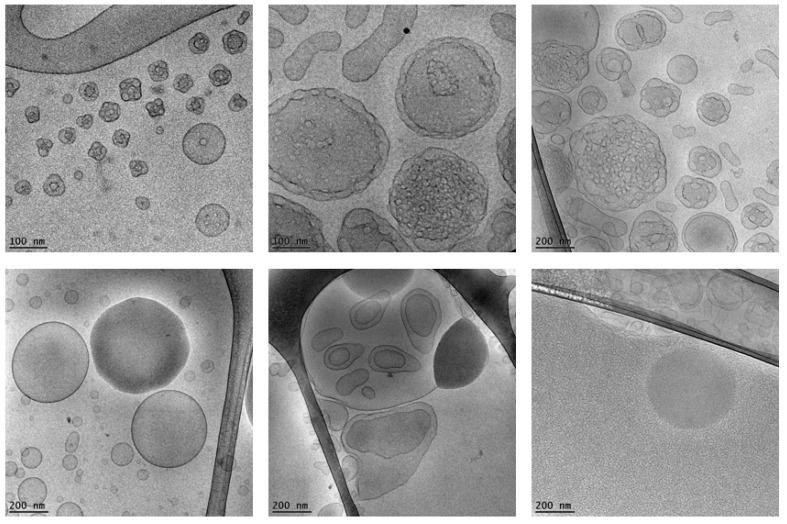
Cryo-TEM images of dispersed liquid crystalline lipid nanoparticles generated by self-assembly of an omega-3 polyunsaturated fatty acid (ω-3 PUFA) and the nonlamellar lipid monoolein. The varying degree of packing and perforation of the bicontinuous lipid membrane yields different types of nano-objects, e.g., small cubosomes, cubosomal intermediates, spongosome particles, swollen sponge-type membranes coexisting with vesicular objects or objects embedding oil-rich domains. The resulting compartmentalized nanocarriers may coencapsulate hydrophobic and hydrophilic guest molecules of interest for combination therapies. (Reprinted from Ref [185]. American Chemical Society Open Access 2018).

Other biometric systems created with bioactive lyotropic lipid self-assembly can also be considered for future studies on neuroprotection and recovery from neuronal damages (Figure 10). For instance, lipid nanoparticles involving bioactive omega-3 polyunsaturated fatty acids (ω-3 PUFAs) have been obtained by self-assembly with the nonlamellar lipid monoolein [185,186]. Multicomponent amphiphilic systems with liquid crystalline self-assembled inner structural organization can serve for the encapsulation of hydrophobic or hydrophilic drugs and natural antiviral compounds for targeting various disease mechanisms as well as pathways favoring recovery from SARS-CoV-2-induced neuronal damage. In examples of multidrug (ω-3 PUFAs, curcumin, and monoglyceride) loading in nanoparticles, curcumin has been considered a drug with antiviral, antioxidant, antimicrobial, antiproliferative, anti-inflammatory, neuroprotective and cardioprotective properties [159]. Monoglycerides have been indicated to have equal capacity for viral inactivation at 5 to 10 times lower concentrations than their corresponding fatty acids. ω-3 PUFAs are adjunctive therapeutics of strong interest for preventive nanomedicine development [159,185]. Recent experimental data have shown that curcumin-loaded lipid nanoparticles may promote BDNF expression and that the ω-3 PUFA content of the nanoparticles may be beneficial for enhancing BDNF activity [159,186].

In recent years, lipid-based therapies have been suggested as an alternative strategy for slowing neurodegeneration and inhibiting neuroinflammation [187,188,189]. PUFA-chain ethanolamine plasmalogens have been described as bioactive lipids and can be 100-fold more powerful in stimulating neurorepair than conventional ω-3 PUFA species [187]. By analyzing the results from clinical and in vitro experiments, it has been concluded that certain plasmalogen lipid derivatives may enhance neurotrophic BDNF signaling and thus promote neurogenesis [189].

**Figure 11:**
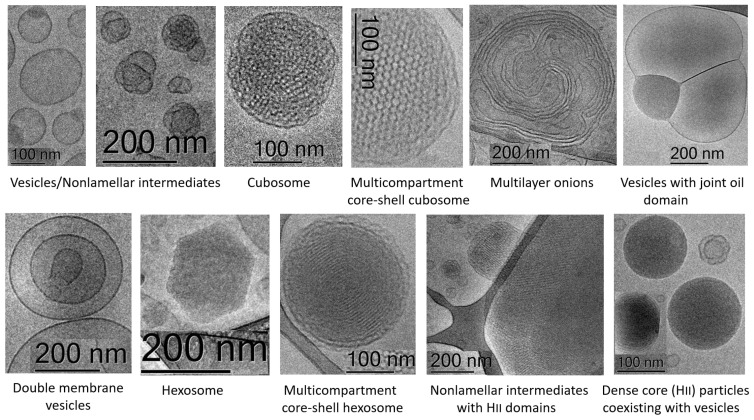
Cryo-TEM images of lipid nanoparticles obtained by self-assembly of custom-synthesized plasmenyl (ether) and ester phospholipids with long PUFA (22:5 n6) chains and the nonlamellar lipid monoolein. The liquid crystalline nanoparticle topologies and the compartmentalized biomimetic supramolecular architectures comprise vesicles, cubosomal intermediates, cubosomes coexisting with vesicles, multicompartment core-shell cubosomes and hexosomes, multilayer onions; vesicles with joint oil domains, double membrane vesicles, nonlamellar intermediates with H_II_ domains, and dense core (H_II_) particles hexosomes coexisting with vesicles. (Reprinted from Ref [190]. Frontiers Open Access 2021).

Based on bioinspiration from biological cubic membranes, self-assembled nanostructures have been designed by mixing synthetic PUFA-chain phospholipids, e.g., plasmenyl phosphoethanolamine (C16:1p-22:5n6 PE), plasmenyl phosphocholine (C16:1p-22:5n6 PC), and DPA-diacyl phosphoinositol (22:5n6-22:5n6 PI) ester, and the nonlamellar lipid monoolein [190]. Various nanoscale object types have been obtained as a result of the structural polymorphism of the investigated lyotropic lipid/DPA-phospholipid mixtures (Figure 11). It has been emphasized that the nanoparticle shape is crucial for drug transport properties. In perspective, further developments should be expected in nanocarrier design for the efficient delivery of CRISPR therapeutics in neurological disorders [191,192,193] as well as in biogenic metal nanoparticles obtained by self-assembly with template agents of natural biological origin, e.g., biogenic silver nanoparticles (AgNPs) [194].

## 6. Conclusions

Biomimetic self-assembly can yield smart nanocarriers providing reduced side effects in therapeutic delivery strategies. The different topologies of the nanocarriers (elongated or spherical nanoparticles with solid or aqueous cores or with inner liquid crystalline membrane organization, nanofiber scaffolds, or gels) may provide different release profiles for encapsulated molecules as well as different resident times at the biological barriers. In recent years, targeted nanocarriers for recombinant growth factors, therapeutic antibodies, enzymes, synthetic peptides, cell-penetrating peptide-drug conjugates, and RNAi sequences have been successfully developed against NDs. Self-assembled nanoscale materials loaded with biotherapeutics can also be used in emerging neuronal regeneration strategies and considered for potential recovery from long-term COVID-19 neuronal dysfunctions. Sustainable BDNF delivery nanoparticles and polymer-based scaffolds have been reported to facilitate neuronal survival and reduce neuronal tissue injury. Safe drug delivery has been achieved to the central and peripheral nervous systems. Enhanced neurogenesis and neuronal survival have been observed both upon growth factor delivery by nanocarriers as well as by exploring the properties of bioactive lipids such as plasmalogens with long PUFA chains. Towards translation into clinics, further research on nanocarrier-mediated drug delivery will be required in the areas of PUFA-chain phospholipids, growth factor therapies, biogenic metal nanoparticles, mRNA therapies, and CRISPR therapeutics.

## Figures and Tables

**Table 1:** Recent examples of nanocarrier-mediated BDNF delivery to the central nervous system.

Nanoformulation	Disease Indications	Administration Route/Model	Outcomes
***Lipid-based nanoparticles***			
Liposomes conjugated with polyethylene glycol (PEG) and transferrin (Tf) as carriers for encapsulated BDNF gene, modified with a glial fibrillary acidic protein promoter (pGFAP) [Tf-pGFAP-BDNF-PEG] or a cytomegalovirus promoter (pCMV) [Tf-pCMV-BDNF-PEG]	Brain injury (degeneration ischemia, and inflammation)	In vivo tail-vein injection	Tf-pGFAP-BDNF-PEG and Tf-pCMV-BDNF-PEG carriers are able to cross the BBB. Predominant expression of BDNF in the cerebral cortex. The Tf-pGFAP-BDNF-PEG group is promoting more significantly the BDNF expression in the cerebral cortex than the Tf-pCMV-BDNF-PEG group [172].
***Polymeric-based nanoparticles and hydrogels***			
PEG-PGA nanoparticle polyion complexes with BDNF	Ischemic stroke	In vivo subcutaneous injection in mice	Reduced tissue injury. Behavioral improvements [165].
BDNF mixed in poly(ethylene glycol)-b-poly(l-glutamic acid) (PEG-PLE) copolymer solution	Neurologic diseases	In vivo Intranasal	Protection of BDNF in the circulation. Better distribution than the native protein. Improved BDNF delivery efficiency [166].
BDNF-loaded micropillarred poly-ε-caprolactone (MP-PCL) or flat PCL (F-PCL) scaffolds	Neuronal lesion	In vitro primary neuronal cultures	Sustained release of BDNF up to 21 days. Increased neuronal survival and synaptic density. Suitable for neural tissue engineering and prosthetics [167].
BDNF in self-assembled IKVAV PA hydrogel	Traumatic spinal cord injuries (TSCI)	In vivo Injection, Spinal cord injury induced using clip compression at T7-T8 vertebral segment	Sustained release of BDNF. Axonal preservation. Astrogliosis decreased at 6 weeks post-injury without inflammation. Locomotor functional recovery failed [168].
BDNF encapsulated in hyaluronic acid hydrogel	Stroke	In vivo Stroke models in mouse (strains C57Bl/6, DBA) and non-human primate (chronic stroke)	Distribution of BDNF-loaded hydrogel from the stroke cavity into the peri-infarct tissue up to 3 weeks compared to 1 week for direct BDNF injection in a mouse model. Recovery of motor function. Migration of immature neurons into the peri-infarct cortex and long-term survival. Released BDNF sufficient for functional recovery from stroke in a non-human primate [169].
BDNF dispersed in a hydrogel, consisting of hyaluronan and methylcellulose, with embedded poly(lactic-co-glycolic acid) nanoparticles	Stroke	In vivo stroke lesions; Stroke-injured rat	Unchanged lesion volume compared to a vehicle group. Synaptophysin expression in homotopic contralesional hemisphere. Better plasticity. [170].
Fibronectin-coated pharmacologically active microcarriers (PAMs) modified with silanized- hydroxypropyl methylcellulose (Si-HPMC) hydrogel for BDNF delivery	Neurological disorders	Human marrow-isolated adult multilineage-inducible (MIAMI) stem cells	The PAMs Si-HPMC hydrogel facilitated the expression of neuronal differentiation markers in MIAMI cells. Improved secretion of growth factors (e.g., b-NGF, HGF, SCF, LIF, SDF-1α, VEGF-A & D) and chemokines (MIP-1α & β, RANTES, IL-8) [171].
PEGylated PAMAM-based nanoparticles	Neurodegenerative diseases	In vitro SH-SY5Y cells	Increased BDNF expression and release for the PEGylated PAMAM nanoparticle group versus the PAMAM-based nanoparticles [176].
***BDNF-mimetic peptide nanofiber scaffolds***			
Self-assemble nanofiber hydrogel including a BDNF mimetic peptide	Peripheral nerve injury	In vivo Rat model	Nerve regeneration and functional recovery observed in a rat model after implantation of nanofiber hydrogels [177].
Nanofibers involving a BDNF mimetic peptide	CNS injuries and diseases	Primary cortical neurons	Neuronal survival and increased functional maturation [178].
***Silica nanoparticles***			
BDNF-loaded porous silica nanoparticles (NPSNPs)	Degeneration of SGNs, inner ear disease	In vitro NIH3T3 fibroblats, SGNs	Sustained BDNF release from amino-modified nanoparticles over 80 days. Cytocompatibility of the NPSNPs with the fibroblasts. Higher survival rate of SGNs in cell cultures as compared to unloaded control NPSNPs [174].

## Data Availability

Not applicable.

## Abbreviations

AD, Alzheimer’s disease; AKAP13, A-kinase anchor protein 13; ApoE, Apolipoprotein E; ALS, amyotrophic lateral sclerosis; ARTN, artemin; BACE1, beta-secretase 1 (beta-site APP cleaving enzyme 1); BBB, blood–brain barrier; BDNF, brain-derived neurotrophic factor; CBP-1, protein cbp-1 acetyltransferase enzyme; CDNF, cerebral dopamine neurotrophic factor; CNS, central nervous system; CNTF, ciliary neurotrophic factor; CSF, cerebrospinal fluid; CT-1, cardiotrophin; DDS, drug delivery system; DYRK1A, dual-specificity tyrosine phosphorylation regulated kinase 1A; ER, endoplasmic reticulum; FGF-1, fibroblast growth factor-1; ER, endoplasmic reticulum; FGF-1, fibroblast growth factor-1; FGF-2, fibroblast growth factor-2; GDNF, glial cell line-derived neurotrophic factor; GCSF, granulocyte colony stimulating factor; GM-CSF, granulocyte-macrophage colony-stimulating factor; IGF-1, insulin-like growth factor 1; IL-6, interleukin 6; LIF, leukemia inhibitory factor; MANF, mesencephalic astrocyte-derived neurotrophic factor; NDs, neurodegenerative diseases; NGCs, nerve guidance conduits; NGF, nerve growth factor; NP, nanoparticles; NRTN, neurturin; NT-3, neurotrophin-3; NT-4, neurotrophin-4; NTFs, neurotrophic factors; PACAP, pituitary adenylate cyclase-activating peptide; PD, Parkinson disease; PDGF, platelet-derived growth factor; PLA, poly(L-lactide); PLGA, poly(lactic-co-glycolic acid); PSPN, persephin; RGD, arginine-glycine-aspartic acid peptide; RNAi, RNA interference; RVG, rabies virus glycoprotein; shRNA, short-hairpin RNA; siRNA, small interfering RNA; SLNs, solid lipid nanoparticles; TrkB, tropomyosin receptor kinase B; UPR, unfolded protein response.
